# Epicardial adipose tissue volume, plaque vulnerability and myocardial ischemia in non-obstructive coronary artery disease

**DOI:** 10.1016/j.ijcha.2023.101240

**Published:** 2023-07-13

**Authors:** Ingela Khan, Caroline A. Berge, Ingeborg Eskerud, Terje H. Larsen, Eva R. Pedersen, Mai Tone Lønnebakken

**Affiliations:** a Department of Clinical Science, University of Bergen, Jonas Lies veg 87, 5021 Bergen, Norway; b Department of Heart Disease, Haukeland University Hospital, Haukelandsveien 22, 5021 Bergen, Norway; c Institute of Biomedicine, University of Bergen, Jonas Lies vei 91, 5009 Bergen, Norway

**Keywords:** Epicardial adipose tissue volume, Myocardial ischemia, Coronary plaque burden, Coronary plaque vulnerability, Cardiac CT

## Abstract

**Background:**

Epicardial adipose tissue (EAT) accumulation has been associated with inflammation, atherosclerosis and microvascular dysfunction. Whether increased EAT volume is associated with coronary plaque vulnerability and demand myocardial ischemia in patients with non-obstructive coronary artery disease (CAD) is less explored.

**Methods:**

In 125 patients (median age 63[58, 69] years and 58% women) with chest pain and non-obstructive CAD, EAT volume was quantified on non-contrast cardiac CT images. EAT volume in the highest tertile (>125 ml) was defined as high EAT volume. Total coronary plaque volume and plaque vulnerability were quantified by coronary CT angiography (CCTA). Demand myocardial ischemia was detected by contrast dobutamine stress echocardiography.

**Results:**

High EAT volume was more common in men and associated with higher BMI, hypertension, increased left ventricular mass index (LVMi), C-reactive protein (CRP) and positive remodelling (all p < 0.05). There was no difference in age, coronary calcium score, total and non-calcified plaque volume or presence of demand myocardial ischemia between groups (all p ≥ 0.34). In a multivariable model, obesity (p = 0.006), hypertension (p = 0.007) and LVMi (p = 0.016) were independently associated with high EAT volume. Including plaque vulnerability in an alternative model, positive remodelling (p = 0.038) was independently associated with high EAT volume.

**Conclusion:**

In non-obstructive CAD, high EAT volume was associated with cardiometabolic risk factors, inflammation and plaque vulnerability, while there was no association with demand myocardial ischemia or coronary plaque volume. Following our results, the role of EAT volume as a biomarker in non-obstructive CAD remains unclear.

## Graphical abstract

**Figure:**
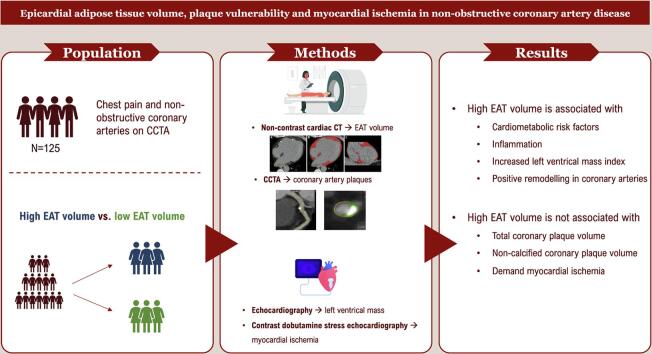

## 1 Introduction

In non-obstructive coronary artery disease (CAD), there is a lack of tailored risk score models and evidence-based recommendations for management [1], [2]. This results in suboptimal identification of high-risk individuals, limiting the possibilities to target prevention and treatment [1], [3], [4]. Myocardial ischemia with non-obstructive coronary arteries (INOCA) has a multifactorial aetiology, including plaque erosions or rupture, microvascular- and endothelial dysfunction, vasospasm or supply demand mismatch [5], [6], [7]. These pathophysiological mechanisms may be detected by advanced non-invasive or invasive imaging. Accordingly, implementation of imaging biomarkers in clinical risk score models has been suggested to improve risk prediction in non-obstructive CAD [8]. Coronary artery plaque burden and particular high-risk plaque features like non-calcified plaque, microcalcification and positive remodelling by coronary computed tomography angiography (CCTA) have been demonstrated to predict myocardial ischemia and risk of cardiovascular (CV) events [2], [9]. Non-invasive or invasive functional testing to directly assess intracoronary flow reserve or microvascular resistance have also been suggested to improve detection of myocardial ischemia and CV risk among patients with non-obstructive CAD [10]. Currently, investigation of patients with non-obstructive CAD is time-consuming, associated with risk of complications and hence is difficult to implement into clinical practise. Therefore, new non-invasive imaging biomarkers are highly warranted to improve the identification of high-risk phenotypes among patients with non-obstructive CAD to individualize treatment and potentially improve prognosis.

Epicardial adipose tissue (EAT) volume, easily assessable by cardiac computed tomography (CT), has been suggested as an imaging biomarker to detect individuals with a high risk of coronary atherosclerosis, microvascular dysfunction and myocardial ischemia [1], [11]. In general, EAT has anti-inflammatory properties and is cardioprotective. However, an increase in EAT volume is associated with release of pro-inflammatory, pro-fibrotic and vasoactive substances directly affecting the coronary arteries and the myocardium through both vasocrine and paracrine pathways [12]. Previous studies and *meta*-analysis have demonstrated that increased EAT volume is associated with CV events, acute myocardial infarction, accelerated atherosclerosis and increased plaque vulnerability [13], [14], [15], [16]. Even though increased EAT volume has been linked to microvascular dysfunction and vasospasm in non-obstructive CAD [17], [18], the association with myocardial ischemia is diverging [19], [20].

We aimed to assess whether measurements of EAT volume can be used as an imaging biomarker to detect high-risk phenotypes with vulnerable plaques and demand myocardial ischemia among patients with non-obstructive CAD. We therefore conducted a cross-sectional, post-hoc analysis to explore the association between EAT volume and cardiometabolic risk factors, inflammation, left ventricular mass, coronary calcium score, demand myocardial ischemia and coronary artery plaque volume and vulnerability.

## 2 Materials and methods

### 2.1 Study design and participants

We included 125 participants from the Myocardial Ischemia in Non-obstructive coronary artery disease (MicroCAD) study. The MicroCAD study is a cross-sectional study, including patients with symptomatic chronic coronary syndrome and non-obstructive CAD (<50% diameter stenosis) by CCTA. The inclusion and exclusion criteria have been described previously [9], [21], [22]. In short, all participants were > 30 years of age and had at least one CV risk factor. Patients with a prior history of obstructive CAD were excluded from the study. All participants signed informed consent to the anonymous use of data for medical research. The study was approved by the Regional Committee for Medical and Health Research Ethics and registered at www.clinicaltrials.gov with trialidentifier NCT01853527.

### 2.2 Cardiovascular risk factors

A quality controlled standardized questionnaire was used to collect information on CV risk factors, known CV disease and medication. In addition, all participants underwent a general clinical examination, including assessments of blood pressure, waist circumference and body mass index (BMI). Fasting blood samples were analysed for serum lipoproteins, hemoglobin A1c (HbA1c) creatinine and C-reactive protein (CRP). Glomerular filtration rate (GFR) was calculated by the Chronic Kidney Disease Epidemiology Collaboration formula [23]. High CRP was adjudicated when CRP ≥ median (≥2 mg/L). BMI was calculated as body weight per height in meters squared. Obesity was defined as BMI ≥ 30 kg/m^2^. Hypertension was defined as known hypertension, use of antihypertensive drugs or office systolic blood pressure ≥ 140 mm Hg and/or diastolic blood pressure ≥ 90 mm Hg [24]. Hypercholesterolemia was defined as use of cholesterol-lowering drugs or total serum cholesterol > 6.5 mmol/l. Diabetes was considered present in subjects with known diabetes or HbA1c ≥ 48 mmol/mol [25]. Participants having a first-degree relative with CAD before the age of 65 years in women and 55 years in men were considered having a family history of premature CAD. Metabolic syndrome was defined as having ≥ 3 of the following criteria: hypertension, diabetes, waist circumference > 102 cm for men and > 88 cm for women, serum triglycerides ≥ 1.7 mmol/l, and HDL < 1.03 mmol/l for men and < 1.3 mmol/l for women.

### 2.3 Non-contrast cardiac computed tomography and coronary computed tomography angiography

Cardiac CT images were acquired using a 2 × 128-slice dual source computed tomography scanner (Somatom Definition Flash, Siemens, Germany) with electrocardiographic-gating in all participants. Recommended guidelines were followed during the procedure [26]. First, participants underwent a non-contrast cardiac CT scan. Thereafter, we performed CCTA with intravenous infusion of non-ionic contrast relative to body weight (80–115 ml iomeprol 400 mg I/ml (Iomeron, Bracco, Milan, Italy)). Participants with heart rate exceeding 60 beats per minute received 1 mg/ml (maximum 20 mg) metoprolol intravenously until heart rate was reduced to ≤ 60 beats per minute. To increase image quality during CCTA, 0.4 mg sublingual nitroglycerin was administered to all participants prior to the procedure.

### 2.4 Epicardial adipose tissue volume

**Fig. 1:**
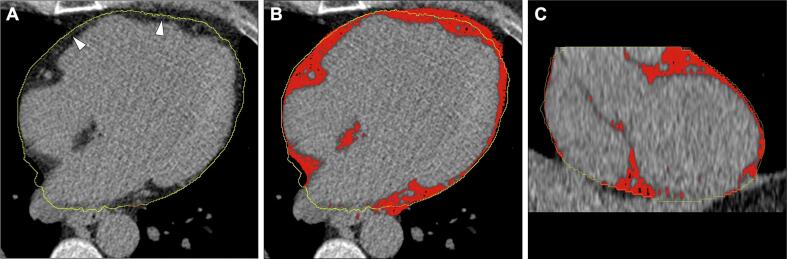
Cardiac CT scans in the axial plane without (A) and with red-colored overlays of fat (B), and in the coronal plane (C). The yellow tracings cover the pericardium (white arrowheads in A) and constitute the borders for EAT measurements.

EAT volume was measured on non-contrast cardiac CT images (Fig. 1). All EAT measurements were performed by a single reader (IK). Images were analysed using a novel semiautomatic software tool (Syngo.via Frontier Cardiac Risk Assessment tool 1.2.0, Siemens, Germany), with an attenuation threshold between −150 and −50 HU. The fat covering the myocardium was automatically traced, and the contour was manually adjusted to cover the pericardium when necessary. The pulmonary artery bifurcation was set as the superior limit, and the level of the posterior descending artery was set as the inferior limit. EAT was quantified as mean volume (ml). Intra-and interobserver reproducibility assessed in five randomly selected patients was good with an intraclass reproducibility of 0.98 and 0.98, respectively.

### 2.5 Coronary calcium score and coronary artery plaque analysis

Coronary calcium score was quantified on non-contrast cardiac CT images by the Agatston method and reported as total calcium score. Non-obstructive CAD was defined as having at least one stenosis in any coronary artery segment with a lumen diameter reduction of < 50%. Advanced coronary artery plaque analysis was assessed by a single experienced reader (IE) in all participants, using a validated semiautomatic analysis software (QAngio CT Research Edition version 3.1.4.2, Medis medical imaging systems, Leiden, The Netherlands) [27], [28]. A 17-segment model from the Society of Cardiovascular Computed Tomography was used to define the coronary segments [9], [29]. Segment involvement score was defined as the total number of coronary artery segments with plaque. Total plaque volume was calculated by subtracting the lumen-volume from the volume of the outer vessel wall. Total plaque volume was divided into total calcified and non-calcified plaque volume, according to plaque composition. Non-calcified plaque was defined as having an attenuation < 350 HU. Vulnerable high-risk plaque was defined as hypodense non-calcified plaque and/or positive remodelling index defined as the luminal diameter maximum stenosis divided by the luminal diameter in a proximal healthy segment > 1.10 [30].

### 2.6 Echocardiography and myocardial contrast dobutamine stress echocardiography

Echocardiography was performed following a standardized protocol and interpreted according to current joint guidelines of the American Society of Echocardiography and the European Association of Cardiovascular Imaging [31], [32]. The same experienced reader (MTL) obtained and analysed all images. In summary, left ventricular (LV) ejection fraction was calculated by Simpsońs biplane method. LV mass (LVMi) was calculated using the Devereux́s equation and indexed for height in m^2.7^ [32], [33], [34]. Relative wall thickness was calculated as the ratio between the left ventricular internal diameter in end-diastole and 2* posterior wall thickness [29]. LV hypertrophy was defined as LVMi exceeding 46.7 g/ m^2.7^ in women and 49.2 g/ m^2.7^ in men [35], [36], and concentric geometry as relative wall thickness ≥ 0.43.

Myocardial contrast dobutamine stress echocardiography with low mechanical index and destruction-replenishment was performed in all participants to evaluate presence and extent of myocardial ischemia, including assessment of regional myocardial perfusion [37]. The contrast agent (SonoVue, Bracco, Milan, Italy) was injected intravenously as a bolus, followed by continuous infusion using a rotating infusion pump (VueJet, Bracco, Milan, Italy). Regional myocardial perfusion was scored at rest and during stress as normal or delayed following a 17-segment LV-model. Peak dobutamine stress was defined as 85% of maximum age-predicted heart rate [37]. Stress-induced myocardial ischemia was defined as delayed contrast-replenishment at peak stress in any of the 17 segments of the LV. Extent of myocardial ischemia was defined as the number of left ventricular segments with delayed contrast-replenishment.

### 2.7 Statistical analysis

The Statistical Package for Social Sciences version 25 (IBM Corporation, Armonk, NY, USA) was used for data management and analysis. Continuous variables are presented as median and interquartile range or as mean ± standard deviation depending on their distribution, while categorical variables are presented as percentages. The participants were divided into two groups according to tertiles of EAT volume, where high EAT volume was adjudicated when the EAT volume was in the highest tertile (≥125 ml).

Independent samples *t*-test, the Mann-Whitney *U* test or Chi-square test was used to compare groups as appropriate following variable characteristics and their distribution. Univariable logistic regression analyses were used to identify covariates of high EAT volume. Variables with significant associations in the univariable analysis and variables of clinical importance were included in multivariable logistic regression models. Results are reported as odds ratio (OR) with their 95% confidence intervals (CI).

## 3 Results

In the total study population, median and interquartile age was 63 (58, 69) years and 58% were women (Table 1). The median and interquartile EAT volume was 104 (77, 141) ml. High EAT volume was more common in men with higher BMI and waist circumference, obesity, hypertension, higher levels of CRP and s-triglycerides, and lower levels of s-HDL (all p < 0.05) (Table 1). Clustering of ≥ 3 CV risk factors were also more common among patients with high EAT volume (Table 1). There was no difference in s-LDL, prevalence of hypercholesterolemia, diabetes, smoking status, family history of premature CAD, HbA1c and estimated GFR between groups (Table 1).

**Table 1:** Clinical characteristics of 1) total study cohort, 2) cohort with low EAT volume and 3) cohort with high EAT volume.

	Total patient cohort n = 125	Cohort with low EAT volume n = 83	Cohort with high EAT volume n = 42	p-value
Age (years), median (IQR)	63 (58, 69)	64 (58, 69)	63 (57, 68)	0.712
Female sex, %	58	67	38	0.002*
BMI (kg/m^2^), median (IQR)	26.7 (24.7, 29.7)	26.2 (23.9, 28.7)	29.0 (26.2, 33.1)	<0.001*
Obesity, %	22	13	38	0.001*
Waist circumference (cm), median (IQR)	97 (90, 103)	94 (86, 101)	103 (100, 111)	<0.001*
Hypertension, %	73	64	93	0.001*
Serum triglycerides (mmol/L), median (IQR)	1.22 (0.87, 1.73)	1.17 (0.85, 1.50)	1.64 (0.91, 2.51)	0.003*
HDL (mmol/L), median (IQR)	1.5 (1.2, 1.8)	1.6 (1.3, 1.9)	1.3 (1.1, 1.6)	<0.001*
LDL (mmol/L), median (IQR)	3.2 (2.4, 4.0)	3.2 (2.4, 4.0)	3.4 (2.5, 4.0)	0.616
Hypercholesterolemia, %	45	42	50	0.406
Diabetes, %	13	14	13	0.909
Current smoker, %	18	17	19	0.809
Family history of premature CAD, %	61	62	59	0.749
HbA1c (mmol/mol), median (IQR)	38 (35, 40)	38 (34, 40)	37 (36, 40)	0.584
Metabolic syndrome, %	30	19	53	<0.001*
Estimated GFR (mL/min/1.73 m^2^), median (IQR)	88 (88, 96)	88 (80, 96)	88 (77, 94)	0.744
≥ 3 cardiovascular risk factors, %	34	28	47	0.041*
CRP ≥ 2 ml/L, %	55%	48%	69%	0.027*

EAT, epicardial adipose tissue; IQR, inter quartile range; BMI, body mass index; CAD, coronary artery disease; GFR, glomerular filtration rate. *p < 0.05.

Increased LVMi and positive coronary artery remodelling were more common among participants with high EAT volume (Table 2). There was no difference in coronary calcium score, segment involvement score, total plaque volume, total non-calcified plaque volume, total calcified plaque volume, and presence and extent of demand myocardial ischemia (Table 2). Furthermore, left atrial volume index, LV ejection fraction, relative wall thickness, LV hypertrophy, LV concentric geometry and LV abnormal geometry did not differ between groups with high or normal EAT volume (Table 2).

**Table 2:** Cardiac CT, CCTA and echocardiographic characteristics of 1) total study cohort, 2) cohort with low EAT volume and 3) cohort with high EAT volume.

	Total patient cohort n = 125	Cohort with low EAT volume n = 83	Cohort with high EAT volume n = 42	p-value
**Cardiac CT**				
Coronary calcium score, median (IQR)	41 (16, 105)	44 (17, 106)	35 (11, 105)	0.601
**CCTA**				
Segment involvement score, median (IQR)	2 (1, 3)	2 (1, 3)	3 (1, 3)	0.521
Total plaque volume (mm^3^), mean ± SD	808 ± 251	828 ± 261	768 ± 226	0.206
Total non-calcified plaque volume (mm^3^), mean ± SD	750 ± 233	764 ± 249	722 ± 197	0.342
Total calcified plaque volume (mm^3^), median (IQR)	27 (9, 64)	28 (10, 91)	23 (9, 50)	0.220
Positive remodelling, %	37	30	50	0.029*
**Echocardiography**				
Presence of myocardial ischemia, %	53	53	52	0.947
Extent of myocardial ischemia, median (IQR)	2 (0, 5)	2 (0, 6)	2 (0, 5)	0.586
Left atrial volume index (ml/m^2^), median (IQR)	14 (12, 17)	15 (12, 17)	14 (12, 16)	0.318
LV ejection fraction (%), mean ± SD	62 ± 7	62 ± 6	61 ± 8	0.197
LVMi (g/m^2.7^), median (IQR)	38.1 (33.1, 45.3)	36.0 (32.7, 43.9)	42.2 (34.8, 47.6)	0.006*
Relative wall thickness, median (IQR)	0.40 (0.34, 0.47)	0.40 (0.35, 0.47)	0.40 (0.33, 0.47)	1.000
LV hypertrophy, %	17	14	21	0.325
LV concentric geometry, %	34	31	40	0.309
LV abnormal geometry, %	44	41	50	0.336

CT, computed tomography; CCTA, coronary computed tomography angiography; LV, left ventricular; LVMi, left ventricular mass index. *p < 0.05.

In univariable logistic regression analysis, female sex, higher BMI, obesity, hypertension, higher s-triglycerides, metabolic syndrome, increased LVMi and positive coronary artery remodeling were associated with high EAT volume (Table 3, univariable). Obesity, hypertension and increase in LVMi remained significantly associated with high EAT volume after multivariable adjustment (Table 3, Model 1). In an alternative multivariable model including plaque vulnerability, sex, hypertension and positive remodelling remained significantly associated with high EAT volume (Table 3, Model 2). There were no associations between high EAT volume and demand myocardial ischemia in uni- or multivariable analyses (Table 3).

**Table 3:** Covariables of high EAT volume in multivariable logistic regression analyses (Model 1 with focus on LV mass index and total plaque volume and Model 2 with focus on plaque vulnerability).

	**Univariable**			**Multivariable**					
**Variables**				**Model 1**			**Model 2**		
	**OR**	**95% CI**	**p**	**OR**	**95% CI**	**p**	**OR**	**95% CI**	**p**
**Age (years)**	0.99	0.95–1.04	0.709	1.00	0.94–1.07	0.967	0.99	0.93–1.05	0.704
**Female sex**	0.30	0.14–0.64	0.002*				0.19	0.07–0.50	<0.001*
**Obesity**	4.03	1.66–9.80	0.002*	5.48	1.63–18.48	0.006*			
**Hypertension**	7.17	2.04–25.22	0.002*	6.38	1.65–24.73	0.007*	10.32	2.45–43.45	0.001*
**LVMi (g/m^2.7^)**	1.06	1.01–1.10	0.012*	1.06	1.01–1.12	0.016*			
**Diabetes**	0.94	0.30–2.91	0.909	0.51	0.12–2.2247	0.371	0.89	0.23–3.50	0.871
**CRP**	2.40	1.10–5.25	0.029*	1.08	0.41–2.86	0.871	1.76	0.69–4.49	0.237
**Total plaque volume (mm^3^)**	0.99	1.00–1.00	0.206	1.00	1.00–1.00	0.531			
**Total non-calcified plaque volume (mm^3)^**	0.99	1.00–1.00	0.340				1.00	1.00–1.00	0.999
**Positive remodelling**	2.32	1.08–4.99	0.031*				2.74	1.06–7.12	0.038*
**Presence of myocardial ischemia**	0.98	0.46–2.10	0.947	0.70	0.27–1.82	0.463	0.51	0.19–1.36	0.513

OR, odds ratio; CI, confidence interval; LVMi, left ventricular mass index. *p < 0.05.

## 4 Discussion

This study, in patients with non-obstructive CAD, demonstrates that high EAT volume is associated with a cardiometabolic high-risk phenotype and inflammation. Metabolic disturbances characterized by central obesity and elevated serum triglycerides, hypertension and increased LVMi, as well as metabolic syndrome, were more common among patients with high EAT volume. In addition, high EAT volume was associated with higher levels of the inflammatory marker CRP. Importantly, patients with high EAT volume also had a higher prevalence of vulnerable high-risk plaque with positive remodelling. However, there were no differences in other established high-risk plaque features, coronary calcium score, plaque burden or presence and extent of stress induced demand myocardial ischemia among patients with high EAT volume and non-obstructive CAD.

EAT accumulation has been associated, not only with metabolic disturbances and inflammation, but also with accelerated atherogenesis. Several previous studies and *meta*-analysis conclude that there is an independent association between EAT volume and the extent and severity of CAD. Both obstructive CAD, vulnerable non-calcified plaque and high-risk plaque features by CCTA have been demonstrated to be more common among patients with higher EAT volume [14], [15], [38]. Plaques with positive remodeling have been demonstrated to have a higher lipid content and macrophage count, features that have been associated with increased vulnerability to plaque rupture and acute cardiovascular events [39]. Our study partly supports the hypothesis that increasing EAT volume is associated with increased plaque vulnerability, by demonstrating an association between positive remodeling and high EAT volume. However, there was no association with coronary artery plaque burden or other high-risk plaque features in our study among patients diagnosed with non-obstructive CAD. These findings are also supported by a recent analysis from the UK Biobank demonstrating that the association between EAT and CAD disappeared after adjusting for confounding CV risk factors [40].

Myocardial ischemia in non-obstructive CAD has a multifactorial etiology involving several different pathophysiological mechanisms. Different disease endotypes including coronary plaque erosion or rupture, microvascular- and endothelial dysfunction, vasospasm and supply demand mismatch have been identified [6]. Accordingly, detection of myocardial ischemia in non-obstructive CAD is challenging, but clinically important and related to prognosis among patients with non-obstructive CAD [41]. EAT volume has been suggested as a new and easily assessable imaging biomarker to be implemented into clinical risk score models to predict risk of myocardial ischemia in patients with non-obstructive CAD. However, contradicting previous *meta*-analysis demonstrating an association between increased EAT volume and myocardial ischemia [14], there was no significant association between EAT volume and presence and extent of myocardial ischemia among our patients with non-obstructive CAD. Nonetheless, multimodality ischemia testing by both non-invasive and invasive modalities are indicated to detect ischemia in non-obstructive CAD. As dobutamine stress echocardiography is mainly detecting wall motion abnormalities in patients with obstructive disease in the epicardial coronary arteries, we performed myocardial perfusion imaging by myocardial contrast dobutamine stress echocardiography to identify demand regional myocardial ischemia associated with microvascular dysfunction [42]. However, to detect myocardial ischemia in patients with suspected coronary vasomotion abnormalities, invasive testing for coronary vasospasms is indicated [43]. In particularly among patients with accumulation of EAT, the associated inflammation and release of vasoactive peptides may cause myocardial ischemia due to vasospasms [44]. Consequently, the lack of association between EAT accumulation and myocardial ischemia in our study should be interpreted with caution, as we only tested for demand myocardial ischemia and no testing for coronary vasospasm was performed.

In clinical practice, recommended risk score models often underestimate CV risk in non-obstructive CAD and dedicated risk score models are warranted [8]. The dual role of EAT in cardioprotection as well as in inflammation and atherosclerosis challenges the potential as a new biomarker of plaque vulnerability and ischemia in non-obstructive CAD. In general, the role of EAT volume in non-obstructive CAD is less explored, however, systemic inflammation has been associated with microvascular dysfunction in obese patients with non-obstructive CAD [45]. Recent studies suggest that both microvascular dysfunction and symptomatic vasospasm are more common in patients with increased EAT thickness assessed by echocardiography [17], [18], however, the studies are few, small and lack standardization. The association between EAT volume and myocardial ischemia therefore needs to be confirmed in larger studies combining measurements of EAT volume and functional coronary artery testing.

## 5 Study limitations

The cross-sectional study design, small sample size and post-hoc analysis of EAT volume limits the statistical power and the possibility to assess causal relationship. There is a need for larger studies to evaluate the performance of EAT volume as a risk predictor in non-obstructive CAD. Our study, including exclusively patients with non-obstructive CAD where the etiology of myocardial ischemia is multifactorial, underlines that the performance of new imaging biomarkers needs to be carefully evaluated for generalizability before implementation into different sub-populations. Furthermore, the prevalence of myocardial ischemia may be underestimated as invasive vasospasm testing was not performed. Future studies should also explore if EAT attenuation and perivascular fat attenuation are better risk predictors in patients with non-obstructive CAD.

## 6 Conclusion

High EAT volume is associated with cardiometabolic risk factors, increased LVMi and inflammation. Even though there is an association between high EAT volume and plaque vulnerability assessed as positive remodelling, there is no association with coronary calcium score, non-calcified plaque or presence and extent of myocardial ischemia. Following our results, the role of EAT volume as a biomarker in non-obstructive CAD remains unclear.

**Funding**

This work was supported by the MedViz Consortium [grant number NA], a collaboration between the University of Bergen, Haukeland University Hospital and Christian Michelsen Research; the Western Norwegian Regional Health Authorities [grant number 911896]; and Hjertefondet, University of Bergen [grant number NA]. None of the sponsors had any influence on the planning, execution, processing of data, writing of the report or in the submission for publication.

## Declaration of Competing Interest

The authors declare that they have no known competing financial interests or personal relationships that could have appeared to influence the work reported in this paper.
